# What Is in a Name?

**DOI:** 10.21470/1678-9741-2024-0221

**Published:** 2025-04-15

**Authors:** Rodrigo Cardoso Cavalcante, Laura Mercer-Rosa, Stephanie M. Fuller

**Affiliations:** 1 Division of Cardiology, Department of Pediatrics, Children's Hospital of Philadelphia, Philadelphia, Pennsylvania, United States of America; 2 Division of Cardiology, Department of Pediatrics, Children's Hospital of Philadelphia and University of Pennsylvania Perelman School of Medicine, Philadelphia, Pennsylvania, United States of America; 2 Division of Cardiothoracic Surgery, Children's Hospital of Philadelphia, Philadelphia, Pennsylvania, United States of America

We congratulate Pinto Junior and co-authors for their important publication drawing attention to the lack of recognition of pediatric congenital heart surgery as a dedicated subspeciality in Brazil^[1]^. The impact of public policies and the historical development of the field of pediatric cardiology are well described. Sadly, this represents almost two decades of delay when compared to the clinical field of pediatric cardiology as a subspecialty in Brazil in 1997, or the recognition of congenital heart surgery as a surgical subspecialty in the United States of America (USA) in 2005.

However, what is in this name? The response must acknowledge those who practice the art of pediatric congenital heart surgery and how one gets to learn it before it is practiced. In a dialogue by Plato, Cratylus^[2]^, we witness a debate on how things should be named:

“Socrates: Therefore, a name is a certain kind of tool meant for teaching and for the disentangling of being, the same way a shuttle is for a weaver’s threads.

Hermogenes: Yes.

S: And the shuttle is for weaving?

H: What else?

S: Therefore, it’s someone who can weave who’ll use a shuttle in a beautiful way, and the beautiful way that belongs to weaving; and it’s someone who can teach who’ll use a name in a beautiful way, and the beautiful is the way that belongs to teaching.

H: Yes.

S: And whose work will the weaver be using beautifully when he uses the shuttle?

H: That of the carpenter.

S: And is everyone a carpenter, or someone who has the art?

H: Someone with the art.

S: And whose work will the hole-driller be using beautifully when he uses the auger?

H: That of the blacksmith.

S: Well, is everyone a blacksmith, or someone who has the art?

H: Someone with the art.”

Who practices the art? The care of a pediatric patient with heart disease encompasses a multidisciplinary team that involves a surgeon, a group of pediatric cardiologists (now in sub-subspecialties), pediatric cardiac anesthesiologists (again, a recognized sub-subspecialty), pediatric residents, perfusionists, nurses, respiratory/occupational/speech therapists, dietitians, pharmacists, psychologists, social workers, and more. With a comprehensive focus on the clinical aspect of pediatric cardiology and its subspecialties, we demonstrate a practice in evolution. But the advancement of the field is also a result of the dedicated pursuits of education, research, and innovation, all of which lead to improved survival and quality of life for this vulnerable population. For example, currently, the rate of prenatal diagnosis is close to 90% in some regions in France^[3]^. Also, the implementation of home monitoring programs for high-risk interstage single ventricle patients has decreased the mortality rate from 12% as reported by the Single Ventricle Reconstruction trial to 8.1% in a British center^[4]^ and to 5.4% at our Philadelphia center^[5]^. These strategies have resulted in a positive impact for patients and families including those at the highest sociodemographic risk^6]^. In the current era, approximately 90% of patients with a congenital heart defect in the USA and Europe reach adulthood^[7]^. These outcomes would not be possible without extraordinary developments including improvements in cardiopulmonary bypass techniques, intraoperative management, and anesthetic practices, intraand postoperative monitoring, and refinement of surgical techniques with innovative device and material development.

How does one learn the art? In the USA, one becomes eligible to train as a pediatric congenital heart surgeon after completing an American College of Graduate Medical Education (ACGME) cardiothoracic surgery residency program (https://www.acgme.org/specialties/thoracic-surgery/program-requirements-and-faqs-and-applications/). There are currently 17 ACGME accredited congenital heart surgery training programs in the USA, all consisting of 24 months duration. During this time, surgeons acquire technical skills to perform complex procedures, such as the Jatene procedure (arterial switch operation), for patients with transposition of the great arteries, and the Norwood procedure, as the first stage of palliation for patients with hypoplastic left heart syndrome (https://www.abts.org/ABTS/Congenital/Congenital_Pathway_1/Congenital%20Index_Cases.aspx). The surgeon also learns the nomenclature of congenital heart diseases (names again!) along with their associated lesions, variable anatomy, and pathophysiology. For each congenital heart lesion, the trained surgeon must understand how the pathophysiology of that condition changes as the patient transitions from fetal life into the newborn period and beyond. This knowledge impacts the timing and indications for surgery, as well as preand postoperative management, and outcomes. This comprehensive training requires significant time, dedication, and personal sacrifice merits recognition.

Therefore, when asked how this group of surgeons should be named, it should be asked instead: what is in a name?
